# Effects of a home-based exercise and physical activity intervention after inpatient rehabilitation on real-world mobility in older adults with cognitive impairment: a secondary analysis of a randomised controlled trial

**DOI:** 10.1093/ageing/afag189

**Published:** 2026-06-26

**Authors:** Christian Werner, Phoebe Ullrich, Letizia Degli Angeli, Rieke Trumpf, Tim Fleiner, Jose L Albites-Sanabria, Luca Palmerini, Bastian Abel, Martin Bongartz, Tobias Eckert, Jürgen M Bauer, Klaus Hauer

**Affiliations:** Geriatric Centre, Medical Faculty Heidelberg, Heidelberg University, Heidelberg, Baden-Württemberg, Germany; Geriatric Centre, Medical Faculty Heidelberg, Heidelberg University, Heidelberg, Baden-Württemberg, Germany; Department of Medical Oncology, Thorax Clinic, Heidelberg University Hospital, Heidelberg, Baden-Württemberg, Germany; Department of Electrical, Electronic, and Information Engineering "Guglielmo Marconi" – DEI, University of Bologna, Bologna, Italy; Department of Geriatric Psychiatry and Psychotherapy, LVR Hospital Cologne, Cologne, North Rhine-Westphalia, Germany; Institute of Movement and Sport Gerontology, German Sport University Cologne, Cologne, North Rhine-Westphalia, Germany; Institute for Geriatric Research, Ulm University Medical Centre, Ulm, Baden-Württemberg, Germany; Agaplesion Bethesda Clinic Ulm, Geriatric Centre Ulm, Ulm, Baden-Württemberg, Germany; Institute of Medical Engineering and Mechatronics, Ulm University of Applied Sciences, Ulm, Baden-Württemberg, Germany; Department of Electrical, Electronic, and Information Engineering "Guglielmo Marconi" – DEI, University of Bologna, Bologna, Italy; Department of Electrical, Electronic, and Information Engineering "Guglielmo Marconi" – DEI, University of Bologna, Bologna, Italy; Geriatric Centre, Medical Faculty Heidelberg, Heidelberg University, Heidelberg, Baden-Württemberg, Germany; Department of Telemedicine and Remote Patient Monitoring, Robert Bosch Hospital, Stuttgart, Baden-Württemberg, Germany; Geriatric Centre, Medical Faculty Heidelberg, Heidelberg University, Heidelberg, Baden-Württemberg, Germany; Geriatric Centre, Medical Faculty Heidelberg, Heidelberg University, Heidelberg, Baden-Württemberg, Germany; Geriatric Centre, Medical Faculty Heidelberg, Heidelberg University, Heidelberg, Baden-Württemberg, Germany; Agaplesion Bethanien Hospital Heidelberg, Heidelberg, Baden-Württemberg, Germany; Geriatric Centre, Medical Faculty Heidelberg, Heidelberg University, Heidelberg, Baden-Württemberg, Germany

**Keywords:** cognitive dysfunction, ageing, motor activity, walking, wearable electronic device, older people

## Abstract

**Background:**

Persistent mobility limitations after inpatient rehabilitation are common in older adults with cognitive impairment (CI). Home-based exercise interventions can improve locomotor capacity during this vulnerable period; however, evidence that they improve real-world mobility is scarce.

**Objective:**

To investigate the effects of a home-based exercise programme combined with physical activity (PA) promotion on real-world digital mobility outcomes (DMOs).

**Design:**

Single-centre, double-blind, randomised, placebo-controlled trial.

**Setting:**

Community.

**Participants:**

104 community-dwelling older adults with CI (82.3 ± 6.0 years; 75% women; Mini-Mental State Examination score 23.2 ± 2.4) recently discharged from inpatient geriatric rehabilitation.

**Methods:**

The intervention group received a 12-week home-based exercise programme combined with behavioural change techniques to promote PA; the control group received a 12-week non-specific home-based placebo motor activity programme. DMOs related to walking amount, pattern and pace were measured over 48 h at baseline, post-intervention and after a 12-week follow-up using a single body-fixed sensor and validated processing algorithms.

**Results:**

Post-intervention, small statistically significant improvements favouring the intervention group were observed in walking pattern and pace outcomes, including longer walking bout (WB) duration, higher walking speed and longer stride length in shorter (10–30 s) WBs, and higher 90^th^ percentile walking speed in WBs >10 s. These improvements were not sustained at the 12-week follow-up. No between-group differences were found for walking amount.

**Conclusion:**

The post-discharge home-based exercise programme combined with PA promotion showed small, short-term improvements in selected real-world walking pattern and pace outcomes in older adults with CI after inpatient rehabilitation; however, these effects were no longer evident at follow-up, and walking amount did not increase.

## Key Points

- The post-discharge home-based exercise and physical activity intervention showed small, short-term improvements in selected real-world walking pattern and pace outcomes.
- The intervention did not increase the amount of real-world walking.
- Improvements in walking pattern and pace were not sustained after follow-up.
- Digital mobility outcomes capturing different domains of real-world walking may complement commonly used walking amount metrics (e.g. step count) by providing more detailed insight into intervention-related changes in daily-life walking behaviour.

## Introduction

Mobility is fundamental for maintaining independence, social participation and quality of life in older age and is a key component of healthy ageing [1]. Older adults with cognitive impairment (CI) often show reduced mobility compared with cognitively healthy peers [2, 3] and face an increased risk of functional decline, falls and institutionalisation [4, 5].

Post-acute care models for older adults, such as inpatient geriatric rehabilitation, aim to restore or maintain functional ability, with mobility as a key domain. However, despite improvements during inpatient rehabilitation [6, 7], mobility limitations often persist after discharge [8, 9].

The post-discharge period is characterised by heightened vulnerability, and impaired mobility during this phase has been associated with functional decline, hospital readmission and mortality [10, 11]. These challenges underscore the need for effective transitional care strategies, particularly for older adults with CI, who are highly prevalent in geriatric rehabilitation settings [12] and experience worse healthcare outcomes after inpatient care than those without CI [13].

Home-based exercise and physical activity (PA) interventions represent a promising strategy to support mobility during the transition from inpatient rehabilitation to community living. Such interventions have been shown to be feasible, safe and effective in improving activities of daily living (ADLs) and locomotor capacity for mobility in community-dwelling older adults with CI [14]. Their home-based nature offers low entry barriers and aligns with many older adults’ preference to remain at home and ‘age in place’ rather than being (re-)admitted to inpatient facilities [15]. However, evidence that such interventions improve actual, real-world mobility, defined as how individuals move within their everyday environments [16], remains limited in older adults with CI after inpatient care [17–20].

Improvements following home-based exercise and PA interventions in older adults with CI have predominantly been demonstrated using standardised performance-based mobility assessments, such as the Short Physical Performance Battery (SPPB), Timed Up and Go (TUG), or 5-Chair Stand Test [14, 17, 19, 21]. These assessments quantify locomotor capacity, referring to an individual’s ability to move under predefined testing conditions [22], but have limited ecological validity for assessing real-world mobility [23].

Although some studies have reported benefits of home-based exercise and PA interventions on real-world mobility using self-reported measures in high-risk older adults [24, 25], including those with CI [17, 18], such measures are prone to recall bias and limited accuracy in older populations, particularly when cognitive function is lower [26]. In contrast, the few post-discharge studies in older adults with CI after inpatient care have not demonstrated increases in objectively measured real-world mobility [19, 20].

Recent advances in wearable sensor technology and analytical methods enable real-world mobility to be assessed using a single body-fixed sensor through so-called digital mobility outcomes (DMOs) [27, 28]. These provide objective, valid measures of real-world walking, capturing not only walking amount but also domains of walking pattern, pace, rhythm and variability [29, 30]. To our knowledge, effects of post-discharge exercise interventions on these walking-related DMOs in older adults, including those with CI, have not yet been investigated.

The randomised controlled HeikE trial evaluated a 12-week home-based exercise programme combined with behavioural change techniques (BCTs) to promote PA in older adults with CI following inpatient geriatric rehabilitation [31]. The intervention was feasible, safe, and cost-effective and led to sustained improvements in locomotor capacity and concerns about falling, as well as short-term benefits in self-reported real-world mobility [18, 19, 32, 33]. However, it did not increase objectively measured real-world mobility amount (active time) [19], and its effects on walking-related DMOs, such as walking amount, pattern, pace, rhythm and variability, remain unknown.

Therefore, this secondary analysis of the HeikE trial investigated the effects of the home-based exercise programme combined with BCTs to promote PA on walking-related real-world DMOs in older adults with CI following inpatient geriatric rehabilitation.

## Methods

### Study design

This study is a secondary analysis of the single-centre, double-blinded, randomised, placebo-controlled HeikE trial (ISRCTN82378327), which evaluated a 12-week home-based exercise programme combined with BCTs to promote PA in community-dwelling older adults with CI after discharge from inpatient geriatric rehabilitation. Assessments were conducted at baseline (T1), at the end of the 12-week intervention (T2), and after a 12-week follow-up (T3) in participants’ homes. The primary outcomes of the parent trial were physical capacity (SPPB) and real-world mobility amount, defined as active time (standing and walking) measured using a chest-attached accelerometer (PAMSys, BioSensics, Cambridge, MA). Detailed information on the trial protocol [31] and the primary and additional secondary outcomes has been reported previously [18, 19, 33]. The HeikE trial was conducted between August 2015 and December 2017 in Heidelberg, Germany. Ethical approval was obtained from the Ethics Committee of the Medical Faculty of Heidelberg University (S-252/2015). The trial was conducted in accordance with the Declaration of Helsinki and its later amendments. Written informed consent was obtained from all participants or their legal representatives prior to enrolment. This secondary analysis followed the Consolidated Standards of Reporting Trials (CONSORT) guidelines for randomised trials, where applicable.

### Participants

Participants were consecutively recruited between August 2015 and April 2017 from inpatient geriatric rehabilitation wards of a German geriatric hospital. Inclusion criteria were age ≥ 65 years; a Mini-Mental State Examination (MMSE) score of 17–26 [34]; ability to walk ≥4 m without a walking aid; discharge from inpatient rehabilitation to private home; residence within 30 km of the study centre; and no participation in another trial. Exclusion criteria were delirium, severe or terminal illness precluding participation, and insufficient German language proficiency.

### Randomisation and blinding

Participants were randomly allocated in a 1:1 ratio to the intervention group (IG) or control group (CG) using sex-stratified urn randomisation, performed after baseline assessment by an independent researcher not involved in recruitment, intervention delivery or outcome assessment. The study was double-blind, with participants being unaware of group allocation, and outcomes assessors were blinded. Both groups followed an identical schedule of home visits and telephone calls to control for potential psychosocial effects of social support.

### Intervention

Participants in the IG received a 12-week home-based exercise programme specifically developed for older adults with CI [17], including progressive balance (side-by-side, semi-tandem, tandem stance) and strength exercises (tiptoe stance, sit-to-stand transfers, stair rise) and an individualised outdoor walking course. It was supplemented by multimodal motivational strategies based on BCTs to promote PA and exercise adherence, including provision of information, goal setting, self-monitoring, social support, barrier identification and problem solving. Participants were instructed to perform the exercises and walking course daily and were supported by sports scientists through five home visits with gradually decreasing frequency and weekly telephone calls. A poster illustrating the exercises and a training manual were provided to facilitate autonomous training. Detailed information on the intervention and adherence has been published previously [31, 32].

### Control

Participants in the CG received a 12-week home-based placebo motor activity programme consisting of a training manual with seated, non-specific flexibility and strength exercises and newsletter-based information on nutrition and relaxation. The programme was supported by the same number and frequency of home visits and telephone calls from the sports scientists.

### Digital mobility outcomes

DMOs were assessed using a single body-fixed sensor (uSense, University of Bologna, Italy; dimensions: 42 × 10 × 68 mm, 36 g) comprising an inertial measurement unit (accelerometer, gyroscope, magnetometer). The sensor was attached to the participants’ lower back (L5) for a battery-constrained monitoring period of 48 consecutive hours during weekdays using waterproof self-adhesive fixing foil. Measurements were conducted on the same days of the week for each participant across all time points (T1-T3). Raw sensor data were standardised according to a predefined data structure described elsewhere [35]. Walking bouts (WBs) were identified using the *Mobilise-D processing pipeline (MobGap)* [36], which applies validated algorithms to derive DMOs [29, 30] across the domains of walking amount, pace, pattern, rhythm and bout-to-bout variability. DMOs derived at the WB level were aggregated to the daily level and averaged across two valid measurement days (≥8 h/day of wear time during waking hours, 07:00–22:00). The walking-related DMO outcomes were not pre-specified in the parent trial protocol. The present main analysis focused on the domains of walking amount, pattern and pace, as these include one of the most commonly studied and clinically interpretable DMOs [28] and have shown stronger evidence for construct validity than rhythm and bout-to-bout variability in mobility-limited older adults [37]: number of steps and walking duration (amount); number of WBs (all, >10 s, >30 s, >60 s), WB duration, 90^th^ percentile (P90) WB duration, and WB duration bout to bout variability (pattern); walking speed in shorter (10–30 s) and longer (>30 s) WBs, P90 walking speed in WBs >10 s and > 30 s, and stride length in shorter (10–30 s) and longer (>30 s) WBs (pace). Additional DMOs related to walking rhythm and bout-to-bout variability were also analysed and are reported in Appendix 1.

### Descriptive measures

Participant characteristics at baseline included age, sex, marital status, education, number of diagnoses, self-reported health status (EQ-5D visual analogue scale [38]), global cognition (MMSE [34]), concerns about falling (Short Falls Efficacy Scale-International [FES-I] [39]), depressive symptoms (Geriatric Depression Scale [40]), fall history in the previous year, walking aid use and locomotor capacity (SPPB [41], supervised gait speed [41], TUG [42]).

### Statistical analysis

Baseline characteristics were summarised as means and standard deviations, medians and interquartile ranges (IQR), or frequencies and percentages (%), as appropriate. Between-group differences in DMOs and changes over time were analysed using linear mixed-effects models for repeated measures with treatment contrasts, with the CG as the reference [43]. The models included group, time and their interaction as fixed effects, with baseline outcome values and sex included as covariates. Participants were included as a random effect, and within-participant correlations across time points were modelled using an autoregressive covariance structure (AR(1)). Models were estimated using restricted maximum likelihood. Analyses followed a modified intention-to-treat (mITT) approach including all randomised participants with at least one valid DMO measurement at any assessment time point, and missing outcome data were handled using maximum likelihood [44]. Model-based estimated marginal means were presented with standard errors, and between-group differences with 95% confidence intervals (CI); associated (unadjusted) *P*-values were also reported. No adjustment for multiple comparisons was applied; results are interpreted as exploratory at *P* < .05. Statistical analyses were performed using R version 4.5.2 (R Foundation for Statistical Computing, Vienna, Austria). The sample size was calculated for the primary outcome of the parent trial (active time), resulting in a target sample of 116 participants after accounting for a 15% dropout rate. Further details are reported in the trial protocol [31].

## Results

### Participant flow and baseline characteristics

**Figure 1:**
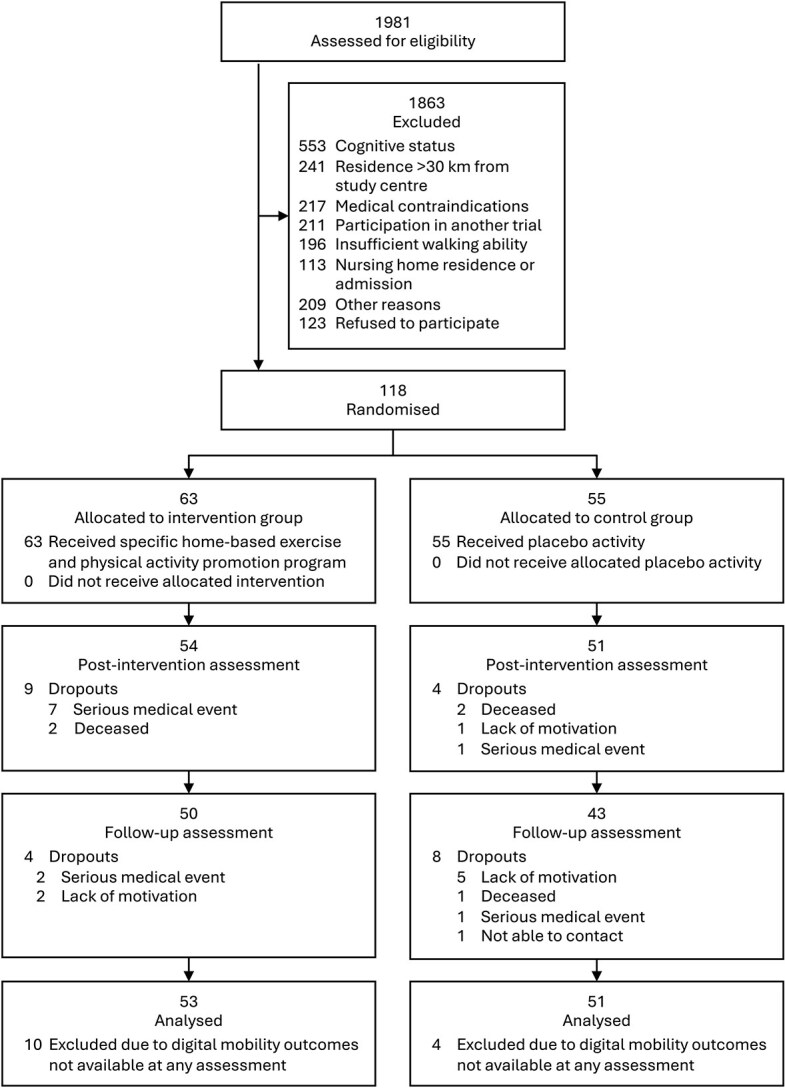
Participant flow for the secondary analysis of digital mobility outcomes.

Of 1981 individuals assessed for eligibility, 1863 were excluded prior to randomisation (Figure 1). A total of 118 participants were randomised to the IG (*n* = 63) or CG (*n* = 55). Fourteen participants (IG: *n* = 10, 15.9%; CG: *n* = 4, 7.3%) provided no valid DMO measurements at any assessment time point and were excluded, yielding a final analytic sample of 104 participants (IG: *n* = 53; CG: *n* = 51). Baseline characteristics were similar between participants included in and excluded from the mITT analysis, except that those without available DMOs were less educated and had lower locomotor capacity (Appendix 2).

Baseline characteristics were well-balanced between the study groups (Table 1). Participants had a mean age of 82.3 ± 6.0 years, and 75% (*n* = 78) were women. Mild-to-moderate CI was observed (mean MMSE score of 23.2 ± 2.4). Locomotor capacity was low, indicated by a mean SPPB score of 5.3 ± 2.3 points, a mean supervised gait speed of 0.51 ± 0.19 m/s, and a median TUG duration of 20.5 s (IQR 15.0–28.5). Walking aid use was highly prevalent (*n* = 84, 80.8%). Participants had multiple comorbid conditions, with a mean of 11.2 ± 4.5 diagnoses.

**Table 1:** Participant characteristics at baseline.

Variables	Total (*n* = 104)^a^	IG (*n* = 53)^a^	CG (*n* = 51)^a^
Age [years], mean ± SD	82.3 ± 6.0	82.2 ± 6.0	82.5 ± 6.0
Women, *n* (%)	78 (75.0)	39 (73.6)	39 (76.5)
Married, *n* (%)	32 (30.8)	14 (26.4)	18 (35.3)
Education, *n* (%)			
Low (≤8 years)	29 (27.9)	14 (26.4)	15 (29.4)
Intermediate (9–12 years)	53 (51.0)	27(50.9)	26 (51.0)
High (≥13 years)	22 (21.2)	12(22.6)	10 (19.6)
Diagnoses, mean ± SD (*n* = 103)^b^	11.2 ± 4.5	11.0 ± 3.7	11.3 ± 5.4
EQ5D VAS score, mean ± SD	55.1 ± 16.1	56.5 ± 17.5	53.7 ± 14.5
MMSE score, mean ± SD	23.2 ± 2.4	23.3 ± 3.0	23.2 ± 2.1
Short FES-I score, median [IQR]	11 [9–14]	11 [9–13]	11 [9–14]
GDS score, mean ± SD	5.3 ± 3.1	5.2 ± 3.0	5.4 ± 3.1
≥1 fall in the previous year, *n* (%)	70 (67.3)	40 (75.5)	30 (58.8)
Walking aid use, *n* (%)	84 (80.8)	43 (81.1)	41 (80.4)
SPPB score, mean ± SD	5.3 ± 2.3	5.5 ± 2.2	5.0 ± 2.4
Supervised gait speed [m/s], mean ± SD (*n* = 100)^c^	0.51 ± 0.19	0.51 ± 0.20	0.51 ± 0.18
TUG [s], median [IQR] (*n* = 102)^d^	20.5 [15.0–28.5]	20.4 [14.7–26.3]	20.6 [15.0–33.9]
**Digital mobility outcomes** (*n* = 100)^e^			
*Amount*			
Step count [#/day], median [IQR]	2206 [904–3876]	2335 [990–4957]	2112 [873–3163]
Walking duration [min/day], median [IQR]	27.8 [13.2–54.3]	29.4 [13.7–64.3]	26.1 [12.9–43.1]
*Pattern*			
Number of WBs [#/day], median [IQR]	144.8 [72.5–215.5]	150.5 [81.5–250.8]	137.5 [63.5–205.0]
Number of WBs >10 s [#/day], median [IQR]	56.0 [26.3–98.3]	52.3 [26.3–118.4]	57.8 [26.5–85.3]
Number of WBs >30 s [#/day], median [IQR]	5.5 [0.8–14.2]	5.8 [0.5–19.5]	4.5 [1.0–10.3]
Number of WBs >60 s [#/day], median [IQR]	0.5 [0.0–3.6]	0.5 [0.0–6.0]	0.0 [0.0–1.8]
WB duration [s], median [IQR]	8.4 [7.4–9.7]	8.0 [7.3–9.6]	8.8 [7.7–9.7]
P90 WB duration [s], median [IQR]	20.8[17.2–26.5]	19.9 [16.7–26.8]	22.3 [17.6–25.3]
WB duration bout-to-bout variability [%], median [IQR]	75.5 [59.6–112.2]	86.8 [61.7–163.9]	70.6 [58.9–97.8]
*Pace*			
Walking speed in shorter (10–30 s) WBs [m/s], mean ± SD (*n* = 98)^f^	0.54 ± 0.08	0.54 ± 0.08	0.54 ± 0.08
Walking speed in longer (>30 s) WBs [m/s], mean ± SD (*n* = 80)^g^	0.60 ± 0.13	0.62 ± 0.12	0.58 ± 0.13
P90 walking speed in WBs >10 s [m/s], mean ± SD (*n* = 98)^f^	0.66 ± 0.13	0.67 ± 0.13	0.64 ± 0.13
P90 walking speed in longer (>30 s) WBs [m/s], mean ± SD (*n* = 80)^g^	0.68 ± 0.17	0.71 ± 0.17	0.65 ± 0.16
Stride length in shorter (10–30 s) WBs [cm], mean ± SD (*n* = 98)^f^	73.1 ± 8.6	73.2 ± 8.4	73.1 ± 9.0
Stride length in longer (>30 s) WBs [cm], mean ± SD (*n* = 80)^g^	81.1 ± 12.9	83.1 ± 12.2	78.9 ± 13.3

Abbreviations: CG, control group; GDS, Geriatric Depression Scale (15-item version); IG, intervention group; IQR, interquartile range; MMSE, Mini-Mental State Examination; P90, 90^th^ percentile; SD, standard deviation; Short FES-I, Short Falls Efficacy Scale-Internationale; SPPB, Short Physical Performance Battery; TUG, Timed Up and Go; VAS, visual analogue scale; WB, walking bout.

^a^Unless otherwise indicated.

^b^Missing data in 1 participant in the CG.

^c^Missing data in 4 participants in the CG.

^d^Missing data in 1 participant in the IG and 1 participant in the CG.

^e^Missing data in 1 participant in the IG and 3 participants in the CG.

^f^Missing data in 1 participant in the IG and 5 participants in the CG.

^g^Missing data in 11 participants in the IG and 13 participants in the CG.

Amount of real-world mobility was low, with a median daily step count of 2206 (IQR 904–3876) and a median daily walking duration of 27.8 min (IQR 13.2–54.3). WBs were predominantly short, with few longer WBs >30 s (median 5.5, IQR 0.8–14.2) and > 60 s (median 0.5, IQR 0.0–3.6). WB duration was also short, with a median of 8.4 s (IQR 7.4–9.7). Real-world walking pace was slow, averaging 0.54 ± 0.08 m/s in shorter (10–30 s) WBs and 0.60 ± 0.13 m/s in longer (>30 s) WBs.

### Intervention effects on digital mobility outcomes

No significant between-group differences were observed post-intervention (T2) or at the 12-week follow-up (T3) for walking amount (step count, walking duration) (Table 2). At T2, small but significant between-group differences favouring the IG were found in DMOs related to walking pattern and pace. WB duration was longer in the IG (mean difference 0.4 s; 95% CI 0.01–0.9; *P* = .047). Walking speed in shorter (10–30 s) WBs (0.02 m/s; 95% CI 0.01–0.03; *P* = .002) and P90 walking speed in WBs >10 s (0.03 m/s; 95% CI 0.01–0.05; *P* = .013) were also higher in the IG. Stride length in shorter (10–30 s) WBs was greater in the IG (1.7 cm; 95% CI 0.3–3.1; *P* = .021). The intervention effects were not sustained at T3, with no significant between-group differences observed for walking amount, pattern or pace. Model-based estimated marginal means for each DMO across all time points by group are provided in Appendix 3.

**Table 2:** Digital mobility outcomes after the intervention (T2) and at the 12-week follow-up (T3) according to group allocation.

Digital mobility outcome	T2				T3			
	IG (mean ± SE)	CG (mean ± SE)	Mean Δ [95% CI]	*P*	IG (mean ± SE)	CG (mean ± SE)	Mean Δ [95% CI]	*P*
**Amount**								
Step count [#/day]	3095 ± 183	3211 ± 185	−116 [−620, 389]	0.650	3033 ± 193	2994 ± 200	39 [−505, 582]	0.888
Walking duration [min/day]	40.1 ± 2.2	39.8 ± 2.3	0.2 [−5.9, 6.4]	0.940	38.9 ± 2.4	37.3 ± 2.4	1.6 [−5.0, 8.2]	0.626
**Pattern**								
Number of WBs [#/day]	167.3 ± 7.3	164.2 ± 7.5	3.2 [−17.1, 23.5]	0.756	168.6 ± 7.7	160.6 ± 8.1	8.0 [−13.8, 29.8]	0.469
Number of WBs >10 s [#/day]	75.2 ± 3.5	68.5 ± 3.6	6.7 [−3.0, 16.3]	0.174	73.0 ± 3.69	66.7 ± 3.9	6.3 [−4.1, 16.7]	0.231
Number of WBs >30 s [#/day]	12.2 ± 1.0	10.8 ± 1.0	1.5 [−1.2, 4.2]	0.281	11.3 ± 1.0	9.6 ± 1.1	1.7 [−1.2, 4.6]	0.248
Number of WBs >60 s [#/day]	3.2 ± 0.4	3.5 ± 0.4	−0.3 [−1.4, 0.9]	0.645	3.3 ± 0.4	2.7 ± 0.4	0.6 [−0.6, 1.9]	0.317
WB duration [s]	9.1 ± 0.2	8.7 ± 0.2	0.4 [0.01, 0.9]	0.047	8.7 ± 0.2	8.5 ± 0.2	0.2 [−0.3, 0.6]	0.504
P90 WB duration [s]	25.1 ± 1.0	23.5 ± 1.1	1.6 [−1.2, 4.5]	0.262	24.7 ± 1.1	23.9 ± 1.2	0.7 [−2.4, 3.8]	0.642
WB duration bout-to-bout variability [%]	104.5 ± 5.4	106.3 ± 5.4	−1.9 [−16.7, 13.0]	0.805	102.8 ± 5.7	104.0 ± 5.9	−1.1 [−17.1, 14.9]	0.891
**Pace**								
Walking speed in shorter (10–30 s) WBs [m/s]	0.56 ± 0.005	0.54 ± 0.005	0.02 [0.01, 0.03]	0.002	0.55 ± 0.005	0.54 ± 0.005	0.00 [−0.01, 0.02]	0.790
Walking speed in longer (>30 s) WBs [m/s]	0.63 ± 0.01	0.60 ± 0.01	0.02 [−0.01, 0.06]	0.127	0.63 ± 0.01	0.62 ± 0.01	0.01 [−0.03, 0.04]	0.665
P90 walking speed in WBs >10 s [m/s]	0.68 ± 0.01	0.66 ± 0.01	0.03 [0.01, 0.05]	0.013	0.66 ± 0.01	0.67 ± 0.01	−0.01 [−0.03 0.01]	0.503
P90 walking speed in longer (>30 s) WBs [m/s]	0.72 ± 0.01	0.70 ± 0.01	0.02 [−0.02, 0.06]	0.341	0.72 ± 0.01	0.70 ± 0.02	0.02 [−0.02, 0.07]	0.263
Stride length in shorter (10–30 s) WBs [cm]	74.9 ± 0.5	73.3 ± 0.5	1.7 [0.3, 3.1]	0.021	73.4 ± 0.5	73.5 ± 0.6	−0.1 [−1.6, 1.4]	0.866
Stride length in longer (>30 s) WBs [cm]	83.5 ± 1.0	81.1 ± 1.1	2.4 [−0.5, 5.3]	0.105	84.0 ± 1.1	81.2 ± 1.1	2.7 [−0.4, 5.8]	0.087

Abbreviations: CG, control group; CI, confidence interval; IG, intervention group; P90, 90^th^ percentile; SE, standard error; WB, walking bout.

Values are model-based estimated marginal means (± SE). Between-group differences (Δ) are model-based estimates with 95% CI, adjusted for baseline values and sex.

## Discussion

This secondary analysis investigated the effects of a 12-week home-based exercise programme combined with BCTs to promote PA on real-world DMOs in older adults with CI following inpatient geriatric rehabilitation. The intervention did not increase real-world walking amount but showed small, short-term improvements in selected walking pattern and pace outcomes after the intervention period. These improvements were not sustained after the 12-week follow-up. To our knowledge, this is the first study to suggest that such an intervention may influence real-world walking pattern and pace in this population.

The absence of intervention effects on walking amount is consistent with previous findings from the HeikE trial, which likewise showed no increase in real-world mobility amount, operationalised as active time (standing and walking) measured with a different wearable sensor [19]. Active time showed strong correlations with step count (*r* = 0.70–0.82) and walking duration (*r* = 0.76–0.84) derived from the uSense sensor across time points (T1–T3), indicating these measures capture closely related aspects of real-world mobility amount. Similarly, another study evaluating a home-based exercise programme combined with BCTs to promote PA in older adults with CI following inpatient care reported no increases in amount-related DMOs (active time) [20]. To our knowledge, only two randomised controlled trials in community-dwelling older adults with cognitive frailty [45] or subjective memory complaints [46] have reported increases in daily step count and/or walking duration after home-based exercise programmes combined with BCTs; however, neither was conducted in a post-discharge context.

Several factors may explain the lack of intervention effects on walking amount. Both groups showed substantial increases in daily step count and walking duration, suggesting that natural recovery and re-engagement with ADLs may have promoted spontaneous increases independent of the intervention. In addition, the non-specific home-based exercises and informational components provided to the CG, combined with structured home visits and telephone calls, may have stimulated activity-related behaviour changes through attention, motivational and social support effects, thereby reducing the intervention-control contrast and potentially contributing to the absence of between-group differences in walking amount.

Low initial locomotor capacity may also have constrained participants’ ability to translate capacity gains into greater walking amount. The mean baseline supervised gait speed of 0.51 m/s suggests substantial restrictions in safe and independent community ambulation [47], which may have made increases in real-world walking amount particularly challenging. This interpretation is supported by previous feasibility findings from the parent HeikE trial showing only moderate adherence to outdoor walking and walking-goal achievement, largely attributed to difficulties integrating walking goals into daily routines due to participants’ physical limitations [32].

Multiple BCTs shown to promote PA in older adults were incorporated into the intervention [48, 49]. Although initial adherence was high, it declined over time alongside the reduced frequency of home visits, as previously reported [32]. Sustained high adherence, potentially facilitated by more frequent and continuous behavioural support and supervision, may have been required to achieve improvements in amount-related DMOs.

The observed small, short-term improvements in selected walking pattern and pace outcomes suggest that the intervention may have influenced how participants walked in their daily environments rather than how much they walked. In this vulnerable population, even small improvements in real-world WB duration, walking speed and stride length may reflect subtle gains in daily-life walking quality, such as walking more efficiently and confidently, with potential relevance for walking safety. This aligns with findings from the HeikE trial demonstrating improvements in supervised gait speed, as well as with broader evidence that home-based exercise programmes can improve walking capacity in older adults with CI [50, 51]. The improvements in locomotor capacity observed in the HeikE trial may thus have carried over into faster real-world walking pace [19], extending previous knowledge on how exercise-related changes may also manifest in everyday walking, whilst the absence of effects on walking amount argues against broader behavioural change.

The magnitude of intervention effects on walking pattern and pace was small, with between-group differences of 0.4 s in WB duration and 0.02–0.03 m/s in walking speed and 2 cm in stride length in shorter WBs. For comparison, 0.05 m/s and 0.10 m/s in supervised gait speed have been reported as small and substantial minimal important differences (MIDs), respectively [52]. However, clinical interpretation is limited because MIDs have not yet been established for real-world DMOs in populations with CI. Recently, MIDs of 0.04 m/s for real-world walking speed in shorter walking bouts and 0.07 m/s for P90 real-world walking speed were reported in a younger, physically and cognitively less impaired sample of community-dwelling older adults after proximal femoral fracture [53]. In our more vulnerable population, the clinical relevance of smaller changes in real-world DMOs remains uncertain and requires further investigation.

The small changes in pace-related DMOs should also be interpreted in light of the high prevalence of walking aid use (80.8%), which may influence real-world walking behaviour. For instance, rollator use has shown to affect the responsiveness of walking capacity measures to intervention-related changes in geriatric patients [54], which may also be relevant for interpretating changes in pace-related real-world DMOs.

Despite these improvements, the lack of sustained effects on walking pattern and pace suggests that short-term intervention benefits may diminish once structured support is withdrawn. This highlights a potential need for longer-term or booster-based strategies, including more continuous and prolonged social support, to help maintain real-world mobility in multimorbid older adults with CI during the post-discharge period.

This study has several limitations. First, DMOs were captured over 48 h due to the limited battery capacity of the uSense sensor, which may not fully reflect habitual real-world walking behaviour. This short monitoring period may have increased measurement variability and reduced estimate reliability. Monitoring periods of ≥3 days are recommended for reliable real-world walking assessment in people with impaired mobility [55], and newer inertial sensors now allow recordings of ˃1 week. Second, the HeikE trial was powered for its primary outcome (active time), not for the DMOs investigated in this secondary analysis; findings should therefore be interpreted as exploratory. Third, only randomised participants with at least one valid DMO measurement were included. Although missing data were handled using maximum likelihood estimation, excluding participants without valid DMO data may have introduced selection bias, limited generalisability and affected the estimated intervention effects, as these participants had lower education and locomotor capacity. Finally, focusing on older adults with CI after inpatient geriatric rehabilitation enhances the clinical relevance for this high-risk group but may limit generalisability to other populations and settings.

In conclusion, this secondary analysis extends the findings of the HeikE trial by showing that a post-discharge 12-week home-based exercise programme combined with BCTs was associated with improvements in selected real-world walking pattern and pace outcomes, but not walking amount, in older adults with CI following inpatient geriatric rehabilitation. Intervention effects were small and not sustained over time, and their clinical relevance remains uncertain. DMOs capturing different domains of real-world walking may complement conventional mobility assessments and commonly used walking amount outcomes (e.g. step count) by providing more detailed insight into intervention-related changes in daily-life walking behaviour.

## Supplementary Material

aa-26-0716-File003_afag189

## Research Data Transparency and Availability

The datasets used and/or analysed during the current study are available from the corresponding author on reasonable request.
