# Supplementary material for: Effects of a home-based exercise and physical activity intervention after inpatient rehabilitation on real-world mobility in older adults with cognitive impairment: a secondary analysis of a randomised controlled trial
Source: Age Ageing. 2026 Jun 26;55(6):afag189. doi: 10.1093/ageing/afag189 (PMC13308959; doi:10.1093/ageing/afag189)
Supplement: aa-26-0716-File003_afag189 [file aa-26-0716-file003_afag189.docx]

Supplementary data

**Effects of a home-based exercise and physical activity intervention after inpatient rehabilitation on real-world mobility in older adults with cognitive impairment: A secondary analysis of a randomised controlled trial**

**Contents list**

**Appendix 1.** Additional digital mobility outcomes after the intervention (T2) and the 12-week follow-up (T3) according to group allocation.

**Appendix 2.** Baseline characteristics of participants with vs. without digital mobility outcomes and included or excluded from the digital mobility outcome analysis.

**Appendix 3.** Model-based estimated marginal means for digital mobility outcomes across all assessment time points according to group allocation.

**Appendix 1.** Additional digital mobility outcomes after the intervention (T2) and the 12-week follow-up (T3) according to group allocation.

| **Digital mobility outcome** | **T2** | | | | **T3** | | | |
| --- | --- | --- | --- | --- | --- | --- | --- | --- |
|  | **IG** (mean ± SE) | **CG** (mean ± SE) | **Mean Δ** [95% CI] | ***p*** | **IG** (mean ± SE) | **CG** (mean ± SE) | **Mean Δ** [95% CI] | ***p*** |
| **Rhythm** |  |  |  |  |  |  |  |  |
| Cadence in all WBs [steps/min] | 88.7 ± 0.5 | 88.0 ± 0.5 | 0.8 [-0.5, 2.0} | 0.236 | 89.3 ± 0.5 | 87.2 ± 0.5 | 2.1 [0.7, 3.4] | 0.003 |
| Cadence in longer (>30 s) WBs [steps/min] | 89.2 ± 0.9 | 88.3 ± 0.9 | 0.9 [-1.6, 3.4] | 0.459 | 89.5 ± 0.9 | 90.5 ± 1.0 | -1.1 [-3.7, 1.6] | 0.428 |
| P90 cadence in longer (>30 s) WBs [steps/min] | 95.8 ± 1.0 | 94.0 ± 1.0 | 1.7 [-1.1, 4.5] | 0.220 | 96.7 ± 1.1 | 95.9 ± 1.1 | 0.8 [-2.2, 3.8] | 0.579 |
| Stride duration in all WBs [s] | 1.28 ± 0.01 | 1.27 ± 0.01 | 0.01 [-0.02, 0.03] | 0.488 | 1.26 ± 0.01 | 1.29 ± 0.01 | -0.03 [-0.06, -0.004] | 0.026 |
| Stride duration in longer (>30 s) WBs [s] | 1.27 ± 0.02 | 1.27 ± 0.02 | -0.01 [-0.05, 0.04] | 0.803 | 1.28 ± 0.02 | 1.27 ± 0.02 | 0.01 [-0.04 0.06] | 0.684 |
| **Bout-to-bout variability** |  |  |  |  |  |  |  |  |
| Walking speed bout-to-bout variability in longer (>30 s) WBs [%] | 14.7 ± 0.9 | 15.0 ± 0.9 | -0.3 [-2.8, 2.2] | 0.795 | 14.8 ± 1.0 | 13.1 ± 1.0 | 1.7 [-1.0, 4.4] | 0.218 |
| Stride length bout-to-bout variability in longer (>30 s) WBs [%] | 11.1 ± 0.7 | 11.4 ± 0.7 | -0.2 [-2.2, 1.8] | 0.795 | 11.4 ± 0.8 | 10.9 ± 0.8 | 0.4 [-1.8, 2.6] | 0.732 |
| Cadence bout-to-bout variability [%] | 10.7 ± 0.2 | 10.9 ± 0.2 | -0.2 [-0.8, 0.4] | 0.547 | 11.3 ± 0.2 | 10.8 ± 0.2 | 0.5 [-0.1, 1.2] | 0.116 |
| Stride duration bout-to-bout variability [%] | 14.8 ± 0.4 | 14.7 ± 0.4 | 0.1 [-0.8, 1.1] | 0.785 | 15.5 ± 0.4 | 14.4 ± 0.4 | 1.2 [0.1, 2.2] | 0.029 |
| CG, control group; CI, confidence interval; IG, intervention group; P90, 90^th^ percentile; SE, standard error; WB, walking bout.  Values are model-based estimated marginal means (± SE). Between-group differences (Δ) are model-based estimates with 95% CI, adjusted for baseline values and sex. | | | | | | | | |

**Appendix 2.** Baseline characteristics of participants with vs. without digital mobility outcomes and included or excluded from the digital mobility outcome analysis.

| **Variables** | **DMOs available** (*n* = 104)^a^ | **DMOs not available** (*n* = 14)^a^ |
| --- | --- | --- |
| Age [years], mean ± SD | 82.3 ± 6.0 | 82.0 ± 6.2 |
| Women, *n* (%) | 78 (75.0) | 12 (85.7) |
| Married, *n* (%) | 32 (30.8) | 4 (28.6) |
| Education, *n* (%) |  |  |
| Low (≤8 years) | 29 (27.9) | 8 (57.1) |
| Intermediate (9-12 years) | 53 (51.0) | 6 (42.9) |
| High (≥13 years) | 22 (21.2) | 0 (0.0) |
| Diagnoses, mean ± SD (*n* = 117)^b^ | 11.2 ± 4.5 | 12.7 ± 3.2 |
| EQ5D VAS score, mean ± SD | 55.1 ± 16.1 | 56.4 ± 14.1 |
| MMSE score, mean ± SD | 23.2 ± 2.4 | 23.9 ± 2.7 |
| Short FES-I score, median [IQR] | 11 [9-14] | 15 [9-17] |
| GDS score, mean ± SD | 5.3 ± 3.1 | 4.9 ± 2.7 |
| ≥1 fall in the previous year, *n* (%) | 70 (67.3) | 9 (64.3) |
| Walking aid use, *n* (%) | 84 (80.8) | 13 (92.9) |
| SPPB score, mean ± SD | 5.3 ± 2.3 | 5.1 ± 2.0 |
| Supervised gait speed [m/s], mean ± SD (*n* = 114)^d^ | 0.51 ± 0.19 | 0.38 ± 0.22 |
| TUG [s], median [IQR] (*n* = 113)^d^ | 20.5 [15.0-28.5] | 23.3 [17.4-26.4] |
| CG, control group; DMO, digital mobility outcome; GDS, Geriatric Depression Scale; IG, intervention group; IQR, interquartile range; MMSE, Mini-Mental State Examination; P90, 90^th^ percentile; SD, standard deviation; Short FES-I, Short Falls Efficacy Scale-Internationale; SPPB, Short Physical Performance Battery; TUG, Timed Up and Go; VAS, visual analogue scale; WB, walking bout.  ^a^ Unless otherwise indicated.  ^b^ Missing data in 1 participant with DMOs.  ^c^ Missing data in 4 participants with DMOs.  ^d^ Missing data in 2 participants with DMOs and 3 participants without DMOs. | | |

**Appendix 3.** Model-based estimated marginal means for digital mobility outcomes across all assessment time points according to group allocation.

| **Digital mobility outcome** | **IG** (mean ± SE) | **CG** (mean ± SE) |
| --- | --- | --- |
| **Amount** |  |  |
| Step count [#/day] |  |  |
| T1 | 2,866 ± 170 | 2,805 ± 175 |
| T2 | 3,095 ± 183 | 3,211 ± 185 |
| T3 | 3,033 ± 193 | 2,994 ± 200 |
| Walking duration [min/day] |  |  |
| T1 | 36.9 ± 2.1 | 36.1 ± 2.1 |
| T2 | 40.1 ± 2.2 | 39.8 ± 2.3 |
| T3 | 38.9 ± 2.4 | 37.3 ± 2.4 |
| **Pattern** |  |  |
| Number of WBs [#/day] |  |  |
| T1 | 162.7 ± 6.8 | 159.7 ± 7.1 |
| T2 | 167.3 ± 7.3 | 164.2 ± 7.5 |
| T3 | 168.6 ± 7.7 | 160.6 ± 8.1 |
| Number of WBs >10 s [#/day] |  |  |
| T1 | 68.4 ± 3.4 | 67.5 ± 3.4 |
| T2 | 75.2 ± 3.5 | 68.5 ± 3.6 |
| T3 | 73.0 ± 3.7 | 66.7 ± 3.9 |
| Number of WBs >30 s [#/day] |  |  |
| T1 | 10.0 ± 0.9 | 9.5 ± 0.9 |
| T2 | 12.2 ± 1.0 | 10.8 ± 1.0 |
| T3 | 11.3 ± 1.0 | 9.6 ± 1.1 |
| Number of WBs >60 s [#/day] |  |  |
| T1 | 2.9 ± 0.4 | 2.5 ± 0.4 |
| T2 | 3.2 ± 0.4 | 3.5 ± 0.4 |
| T3 | 3.3 ± 0.4 | 2.7 ± 0.4 |
| WB duration [s] |  |  |
| T1 | 8.7 ± 0.2 | 8.7 ± 0.2 |
| T2 | 9.1 ± 0.2 | 8.7 ± 0.2 |
| T3 | 8.7 ± 0.2 | 8.5 ± 0.2 |
| P90 WB duration [s] |  |  |
| T1 | 23.2 ± 1.0 | 23.1 ± 1.0 |
| T2 | 25.1 ± 1.0 | 23.5 ± 1.1 |
| T3 | 24.7 ± 1.1 | 23.9 ± 1.2 |
| WB duration bout-to-bout variability [%] |  |  |
| T1 | 100.5 ± 5.0 | 93.8 ± 5.1 |
| T2 | 104.5 ± 5.4 | 106.3 ± 5.4 |
| T3 | 102.8 ± 5.7 | 104.0 ± 5.9 |
| **Pace** |  |  |
| Walking speed in shorter (10-30 s) WBs [m/s] |  |  |
| T1 | 0.54 ± 0.004 | 0.54 ± 0.005 |
| T2 | 0.56 ± 0.005 | 0.54 ± 0.005 |
| T3 | 0.55 ± 0.005 | 0.54 ± 0.005 |
| Walking speed in longer (>30 s) WBs [m/s] |  |  |
| T1 | 0.60 ± 0.01 | 0.60 ± 0.01 |
| T2 | 0.63 ± 0.01 | 0.60 ± 0.01 |
| T3 | 0.63 ± 0.01 | 0.62 ± 0.01 |
| P90 walking speed in WBs >10 s [m/s] |  |  |
| T1 | 0.66 ± 0.01 | 0.66 ± 0.01 |
| T2 | 0.68 ± 0.01 | 0.66 ± 0.01 |
| T3 | 0.66 ± 0.01 | 0.67 ± 0.01 |
| P90 walking speed in longer (>30 s) WBs [m/s] |  |  |
| T1 | 0.69 ± 0.01 | 0.69 ± 0.01 |
| T2 | 0.72 ± 0.01 | 0.70 ± 0.01 |
| T3 | 0.72 ± 0.01 | 0.70 ± 0.02 |
| Stride length in shorter (10-30 s) WBs [cm] |  |  |
| T1 | 73.4 ± 0.5 | 73.5 ± 0.5 |
| T2 | 74.9 ± 0.5 | 73.3 ± 0.5 |
| T3 | 73.4 ± 0.5 | 73.5 ± 0.6 |
| Stride length in longer (>30 s) WBs [cm] |  |  |
| T1 | 81.7 ± 0.9 | 81.1 ± 1.0 |
| T2 | 83.5 ± 1.0 | 81.1 ± 1.1 |
| T3 | 84.0 ± 1.1 | 81.2 ± 1.1 |
| **Rhythm** |  |  |
| Cadence in all WBs [steps/min] |  |  |
| T1 | 87.8 ± 0.4 | 87.8 ± 0.4 |
| T2 | 88.7 ± 0.5 | 88.0 ± 0.5 |
| T3 | 89.3 ± 0.5 | 87.2 ± 0.5 |
| Cadence in longer (>30 s) WBs [steps/min] |  |  |
| T1 | 88.2 ± 0.8 | 87.9 ± 0.9 |
| T2 | 89.2 ± 0.9 | 88.3 ± 0.9 |
| T3 | 89.5 ± 0.9 | 90.5 ± 1.0 |
| P90 cadence in longer (>30 s) WBs [steps/min] |  |  |
| T1 | 94.6 ± 0.9 | 94.2 ± 1.0 |
| T2 | 95.8 ± 1.0 | 94.0 ± 1.0 |
| T3 | 96.7 ± 1.1 | 95.9 ± 1.1 |
| Stride duration in all WBs [s] |  |  |
| T1 | 1.27 ± 0.01 | 1.28 ± 0.01 |
| T2 | 1.28 ± 0.01 | 1.27 ± 0.01 |
| T3 | 1.26 ± 0.01 | 1.29 ± 0.01 |
| Stride duration in longer (>30 s) WBs [s] |  |  |
| T1 | 1.29 ± 0.01 | 1.30 ± 0.01 |
| T2 | 1.27 ± 0.02 | 1.27 ± 0.02 |
| T3 | 1.28 ± 0.02 | 1.27 ± 0.02 |
| **Bout-to-bout variability** |  |  |
| Walking speed bout-to-bout variability in longer (>30 s) WBs [%] |  |  |
| T1 |  |  |
| T2 | 14.7 ± 0.9 | 15.0 ± 0.9 |
| T3 | 14.8 ± 1.0 | 13.1 ± 1.0 |
| Stride length bout-to-bout variability in longer (>30 s) WBs [%] |  |  |
| T1 | 11.4 ± 0.7 | 11.1 ± 0.7 |
| T2 | 11.1 ± 0.7 | 11.4 ± 0.7 |
| T3 | 11.4 ± 0.8 | 10.9 ± 0.8 |
| Cadence bout-to-bout variability [%] |  |  |
| T1 | 11.0 ± 0.2 | 11.0 ± 0.2 |
| T2 | 10.7 ± 0.2 | 10.9 ± 0.2 |
| T3 | 11.3 ± 0.2 | 10.8 ± 0.2 |
| Stride duration bout-to-bout variability [%] |  |  |
| T1 | 15.2 ± 0.3 | 15.2 ± 0.3 |
| T2 | 14.8 ± 0.4 | 14.7 ± 0.4 |
| T3 | 15.5 ± 0.4 | 14.4 ± 0.4 |
| CG, control group; CI, confidence interval; IG, intervention group; P90, 90^th^ percentile; SE, standard error; WB, walking bout. | | |
